# Cow urine distillate as bioenhancer

**DOI:** 10.4103/0975-9476.74089

**Published:** 2010

**Authors:** Gurpreet Kaur Randhawa

**Affiliations:** *Department of Pharmacology, Government Medical College, Amritsar, Punjab - 143 001, India. kullar.g@gmail.com*

Sir,

In the *“Journal of Ayurveda and Integrative Medicine”* April 2010, 1(2), the article on “Bioenhancers – Revolutionary concept in market” is very aptly written. In addition to the herbal bioenhancers elucidated in the article, I would like to add that cow urine distillate/concentrate *(Kamdhenu Ark)* shares this property too.

Cow *(Bos indicus)* urine/*gomutra* has been elaborately explained in Ayurveda and described in *“Sushruta Samhita”, “Ashtanga Sangraha”* and other Ayurvedic texts as an effective medicinal substance/secretion of animal origin with innumerable therapeutic properties.[1] Bhav Prakash Nighantu describes *gomutra* as the best of all types of animal urine (including human) and enumerates its various therapeutic uses.[2] Persons who drink *gomutra* regularly are said to live a healthy life, remaining unaffected by the vagaries of old age, even at age 90.[3] *Gomutra* is called *“Sanjivani”* and “*Amrita*” in Ayurveda. In addition, it has applications as a biopesticide in organic farming along with cow dung, cow’s milk and other herbal ingredients.

*Gomutra* is not a toxic waste material. 95% of it is water, 2.5% consists of urea, and the remaining 2.5% is a mixture of minerals, salts, hormones and enzymes.[4] *Gomutra* exhibits the property of *Rasayana tattwa* responsible for modulating various bodily functions, including immunity. It augments B- and T-lymphocyte blastogenesis; and IgG, IgA and IgM antibody titers in mice. It also increases secretion of interleukin-1 and interleukin-2,[5] phagocytic activity of macrophages, and is thus helpful in the prevention and control of infections. Antimicrobial and germicidal properties of *gomutra* are due to the presence of urea (strong effect), creatinine, swarn kshar (aurum hydroxide), carbolic acid, other phenols, calcium and manganese; its anticancer effect is due to uric acid’s antioxidant property and allantoin; immunity is improved by swarn kshar; and wound healing is promoted by allantoin. Cardiovascular health is maintained by a number of its components: kallikrein is a vasodilator; the enzyme urokinase acts as a fibrinolytic agent; nitrogen, uric acid, phosphates and hippuric acid act as diuretic agents; ammonia maintains the integrity of blood corpuscles; nitrogen, sulfur, sodium and calcium components act as blood purifiers; while iron and erythropoietin stimulating factor maintain hemoglobin levels. Renal health is maintained by nitrogen, which acts as a renal stimulant, and urinary components which act as diuretic agents. Its antiobesity effect is due to the presence of copper ions; calcium promotes skeletal/bone health. Aurum hydroxide and copper act as antidotes for various poisons in the body.[6]

Certain poisons can be refined and purified if soaked in *gomutra* for 3 days. For example, *Dhatura (Dhatura metel)* seeds (with shell peeled off) are considered purified after soaking in *gomutra* for 12 hours. Cow urine can be used for purification of guggul *(Comniphera mukul), loha* (iron) and bhalataka *(Semecarpus anacardium)*, detoxification of aconite *(Aconitum napellus)* and also for purification and detoxification of silver.[7]

Bioenhancing is one of its many properties.[8] Cow urine distillate is more effective as a bioenhancer than cow urine, and increases the effectiveness of antimicrobial, antifungal and anticancer drugs.[9] It also increases the activity of gonadotropin releasing hormone conjugate with bovine serum albumin (GnRH-BSA) and zinc.[10]

Cow urine has bioenhancing activity for Rifampicin, the front-line anti-tubercular drug used against tuberculosis, increasing its action up to sevenfold against *Escherichia coli*, and up to 11-fold against Gram-positive bacteria. Cow urine distillate enhances the transport of antibiotics, e.g., Rifampicin, Tetracycline, and Ampicillin, across the gut wall as well as across artificial membranes. Transport enhancement varies from approximately twofold to sevenfold.[11]

The GnRH–BSA conjugate has a deleterious effect on reproductive hormones and estrous cycles of female mice; cow urine concentrate acts as a bioenhancer of immunization efficacy to modulate these effects.[10]

Cow urine exhibits antitoxic activity against cadmium chloride and can be used as a bioenhancer for zinc, Zn^2+^. Mature male mice, *Mus musculus*, exposed to cadmium chloride only, showed 0% fertility rate. However, the animals given a combination of cadmium chloride + cow urine + zinc sulfate showed 90% fertility rate with 100% viability and lactation indices. Besides this, the fertility index was also found to be 88% in the group treated with cadmium chloride and cow urine.[12]

Cow urine has been granted US Patents (No. 6,896,907 and 6,410,059) for its medicinal properties, particularly as a bioenhancer and as an antibiotic, antifungal and anticancer agent. With regard to the latter, it has been observed to increase the potency of “Taxol” (paclitaxel) against MCF-7, a human breast cancer cell line, in *in vitro* assays (US Patent No. 6,410,059).

These milestone achievements highlight the potential role of cow urine in treatment of bacterial infections and cancer, and demonstrate that cow urine can enhance the efficacy and potency of other drugs.

## References

[1] Prashith Kekuda TR, Nishanth BC, Praveen Kumar SV, Kamal D, Sandeep M, Megharaj HK (2010). Cow urine concentrate: A potent agent with antimicrobial and anthelmintic activity. J Pharm Res.

[2] Pandey GS, Chunekar KC (2009). Bhav Prakash Nighantu (Indian Materia Medica) of Sri Bhavamisra (c.1600-1600 AD) - Ath Mutravargh.

[3] Shukla AV, Tripathi RD (1997). Caraka Samhita of Agnivesh.

[4] Bhadauria H (2002). Cow Urine- A Magical Therapy. Vishwa Ayurveda Parishad, 71-74. Int J Cow Sci.

[5] Chauhan RS (2004). Panchagavya Therapy (Cow pathy)- Current status and future directions. Indian Cow.

[6] Jain NK, Gupta VB, Garg R, Silawat N (2010). Efficacy of cow urine therapy on various cancer patients in Mandsaur district, India: A survey. Int J Green Pharm.

[7] Misra BS, Shastri KA, Lochan K, Choudhary AK (2006). Bhaisajyaratnavali of Govinda Dasji.Vol.

[8] Chauhan RS, Garg N (2003). Cow Therapy as an alternative to antibiotics.

[9] Chawla PC (2010). Risorine - A Novel CSIR Drug Curtails TB Treatment, CSIR News. March.

[10] Ganaie JA, Shrivastava VK (2010). Effects of gonadotropin releasing hormone conjugate immunization and bioenhancing role of Kamdhenu ark on estrous cycle, serum estradiol and progesterone levels in female Mus musculus. Iran J Reprod Med.

[11] http://www.patentstorm.us/patents/6896907/description.html.

[12] Khan A, Srivastava V (2005). Antitoxic and Bioenhancing role of Kamdhenu ark2(Cow urine distillate) on fertility rate of male mice (mus musculus) affected by cadmium Chloride Toxicity. Int J Cow Sci.

